# Hypopituitarism After Traumatic Brain Injury

**DOI:** 10.7759/cureus.4163

**Published:** 2019-03-01

**Authors:** Sanjiv Gray, Tracy Bilski, Beatrice Dieudonne, Saqib Saeed

**Affiliations:** 1 Surgery, University of Central Florida, Orlando, USA; 2 Surgery, University of South Florida Morsani College of Medicine, Kissimmee, USA; 3 Surgery, Harlem Hospital Center, New York, USA

**Keywords:** hypopituitarism, traumatic brain injury, head injury, neuroendocrine dysfunction, adrenal insufficiency, diabetes insipidus, central diabetes insipidus

## Abstract

Acquired hypopituitarism is associated with traumatic brain injury. This neuroendocrine dysfunction can cause both short-term and long-term morbidity resulting in a cognitive, physiological, and behavioral decline, which increases the burden of the disease and the cost of care. Data in the trauma literature is derisory on this subject. The aim of this review is to edify clinicians on this condition, outline the screening criteria and methods for hypopituitarism after traumatic brain injury, and bring awareness to the chronic effects.

## Introduction and background

Traumatic brain injury (TBI) has a significant impact on our population with an incidence of approximately 2.8 million cases per year in the United States and approximately 50,000 deaths annually. After exsanguination, TBI has been found to be the leading cause of death in the trauma population; among the survivors, 80,000 - 90,000 will experience the onset of long-term disability [1]. The leading causes of TBI are falls (54 - 79%), being struck by or against an object (15%), and motor vehicle collisions (15%). Between 2007 and 2013, TBI-related emergency department visits increased by 47%, hospitalization rates decreased by 2.5%, and death rates decreased by 5% [1]. Most cases are mild but approximately 20 - 40% are classified as moderate or severe. With improvements in the trauma system, including prehospital care, trauma resuscitation, and development of TBI protocols, more patients are surviving to discharge. These survivors often have physical, cognitive, and behavioral alterations. Acute hypopituitarism can affect the patient's hemodynamics and can have an influence on the long-term recovery from the TBI. This review aims to highlight an increasingly recognized entity, which can have an impact on the functional outcomes of TBI.

## Review

Cyran et al. [2] first described hypopituitarism after TBI in 1918, and in recent years, there have been multiple descriptions in the neurology, endocrine, and physiatry literature. Clinicians need to be aware of these post-TBI endocrinopathies and provide appropriate referral to an endocrinologist or internist for further testing and management. Hypopituitarism is underdiagnosed in the TBI population as signs and symptoms are vastly non-specific in these critically ill trauma patients. Pituitary failure was previously thought to be rare, but recent literature reviews show the prevalence of hypopituitarism ranges from 15% to 90% [3-8]. In a systematic review, Schneider et al reported a pooled prevalence of 27.5% for hypopituitarism after TBI with the prevalence of anterior pituitary dysfunction ranging from 15% to 68% across all studies in the review [8]. This would make TBI the most common cause of acquired hypopituitarism. Several factors contribute to the pathophysiology of hypopituitarism after TBI, such as a direct pituitary gland/stalk injury or ischemia/infarction of the pituitary gland [9-10], which can be attributed to venous infarction of the long hypophyseal portal veins. Secondary insults arise from hypoxia, hypotension, anemia, raised intracranial pressure, and reduced cerebral perfusion pressure. Tanriverdi et al. suggest that autoimmunity may cause neuroendocrine dysfunction with the presence of anti-pituitary antibodies up to three years after diagnosis [11-12]. Autoimmunity may result from the disruption of the blood-brain barrier, which exposes brain proteins to the systemic circulation. Magnetic resonance imaging (MRI) is the preferred method to assess the pituitary gland post-injury [13].

Understanding pituitary hormonal function 

Understanding the function of the various pituitary hormones will aid the clinician in screening for symptoms of deficiency of specific hormones. Growth hormone (GH) is important for microtubular regeneration, lipid metabolism, and dendritic growth and regrowth. Insulin-like growth factor 1 (IGF-1) affects the functional use of glucose in the brain. IGF-1 depletion causes disruption in lipid and microtubule metabolism, leading to impaired neuronal, somatic, and dendritic growth. Functions mediated by GH receptors in the hippocampal area may be involved in the hormone’s action on memory and cognitive function [14]. Adrenocorticotropic hormone (ACTH) and cortisol deficiency result in fatigue, weakness, and inability to respond to physiological stress. In addition, mood disorders, decreased memory, and frank psychosis can be seen with chronic cases of cortisol deficiency. Decreases in thyroid function lead to a decrease in the basal metabolic rate. This combination could lead to symptoms of physical fatigue. Follicle-stimulating hormone (FSH) and luteinizing hormone (LH) play integral parts in the production of the sex hormones, estrogen, and testosterone. Besides the effects on reproduction, menses, libido, fertility, pregnancy, and maintenance of sexual characteristics, deficiencies in these hormones have also been linked to decreases in bone and muscle mass [15].

The degree of hypopituitarism is determined by the number of axes involved. Growth hormone deficiency is the most commonly reported deficiency in severe and moderate TBI with a prevalence of 2% to 66% [4, 6, 16]. Adrenal deficiency ranges from 0 to 60%, hypothyroidism from 0 to 29%, central hypogonadism from 0 to 29%, and hyperprolactinemia from 0 to 48%. The wide ranges and variability are explained by the lack of screening guidelines, different intervals from TBI occurrence to screening, varying severity of head injury, different testing methods, and different study designs [17]. Most of the literature includes case studies, retrospective studies, and cross-sectional studies. Adrenal insufficiency (AI) and central diabetes insipidus (DI) are common in the acute phase but tend to resolve. Hyperprolactinemia cases are mild and are typically clinically insignificant [18].

Screening and diagnosis

**Figure 1:**
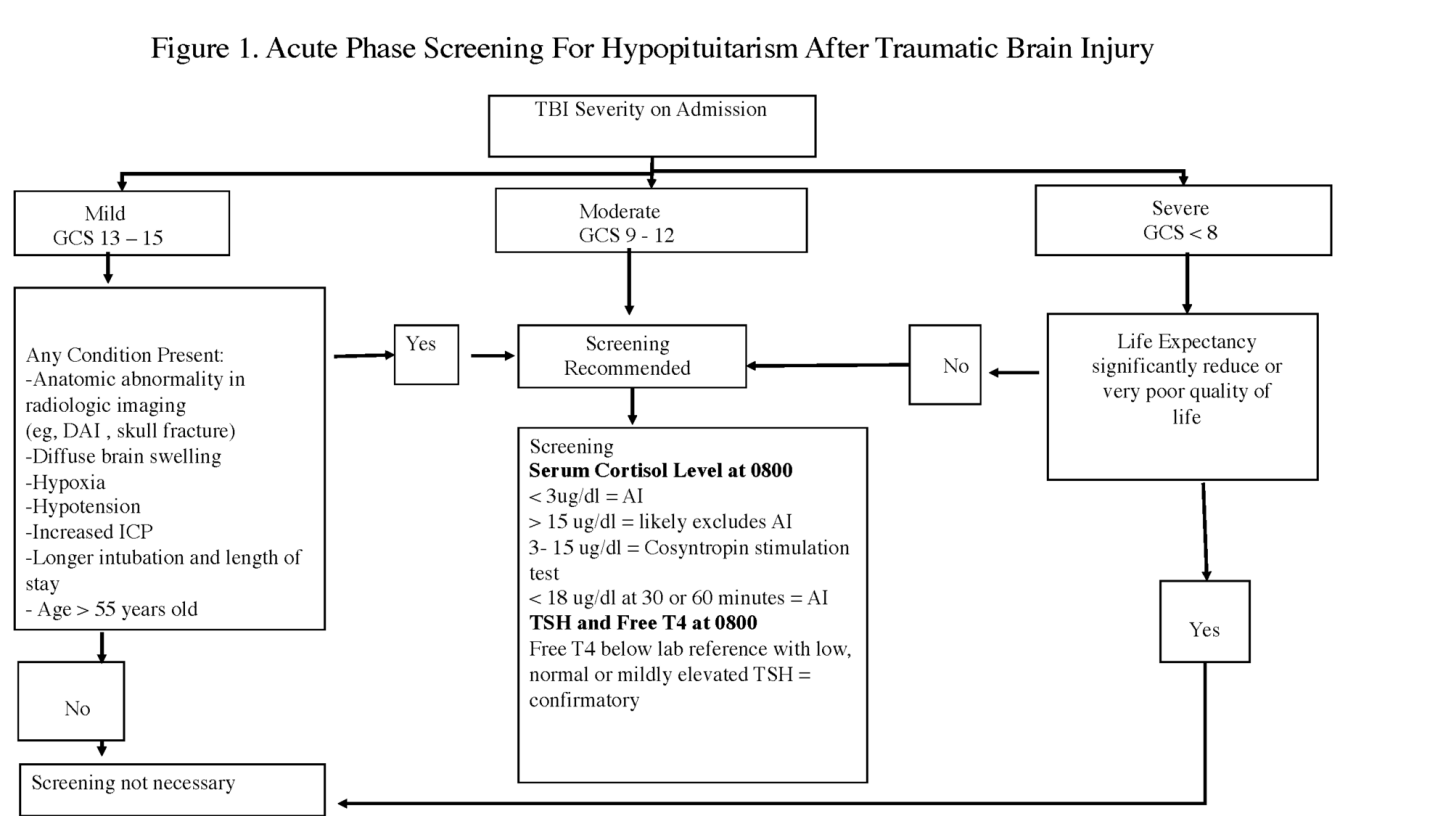
Acute Phase Screening for Hypopituitarism After Traumatic Brain Injury AI - adrenal insufficiency; DAI - diffuse axonal injury; GCS - Glasgow Coma Scale; ICP - intracranial pressure; TBI - traumatic brain injury; TSH - thyroid stimulating hormone; ug/dl - microgram per deciliter

The optimal time for screening is still debatable as pituitary dysfunction during the acute phase of TBI to three weeks does not necessarily lead to long-term hypopituitarism [8]. Schneider et al. showed that hypopituitarism is found in 56% of TBI patients at three months but only in 36% at one year. The prevalence of hypopituitarism in Schneider's series for cases of severe, moderate, and mild TBI were 35.3%, 10.9%, and 16.8%, respectively [19]. In addition, trauma patients have hormonal changes and systemic inflammatory response syndrome with findings similar to pituitary failure. There is also increased cortisol, growth hormone, prolactin, and vasopressin as part of the stress response and acute adaptive response to injury [20]. These levels are also affected by medications, surgery, and drug intoxication. Pituitary function during this time is noted to be variable with either worsening or improving function. This has been attributed to the revascularization of the cellular pituicyte [21]. In a consensus statement, Ghigo et al. maintained that routine basal hormonal testing should be performed on any patient who has been hospitalized with a TBI and has symptoms, such as hyponatremia and hypotension, to rule out adrenal insufficiency, which can be life-threatening (Figure 1) [22]. They also recommended that patients who sustained a mild TBI and are symptomatic or admitted greater than 24 hours and those with moderate and severe TBI should undergo a baseline hormonal evaluation at three and 12 months post-head injury. All post-TBI patients with any signs or symptoms of hypopituitarism should undergo hormonal testing without further delay, even if greater than one year from the injury, as it is unlikely that any hormonal deficit would be transient after that period of time. Lorenzo et al. [23] proposed screening at one year after TBI, especially for gonadal and somatotropic axes, while acutely replacing other needed hormones.

Recommendations of an expert panel assembled by the Defense Centers of Excellence (DCoE) for Psychological Health and Traumatic Brain Injury to address neuroendocrine dysfunction in mild TBI (Glasgow Coma Scale (GCS): 12-15) was released in December 2012 to encourage primary care providers to consider screening patients with persistent symptoms after TBI and even up to 36 months post-injury [24]. Other studies have shown that ongoing dynamic hormonal changes persist up to three years in mild TBI. 

Patients in a persistent vegetative state [25] or with a low life expectancy will not benefit from hormone replacement; therefore, screening is not recommended. In addition, some patients with mild TBI or repetitive head injury maybe develop isolated hypopituitarism; therefore, vigilance is needed [26]. 

**Figure 2:**
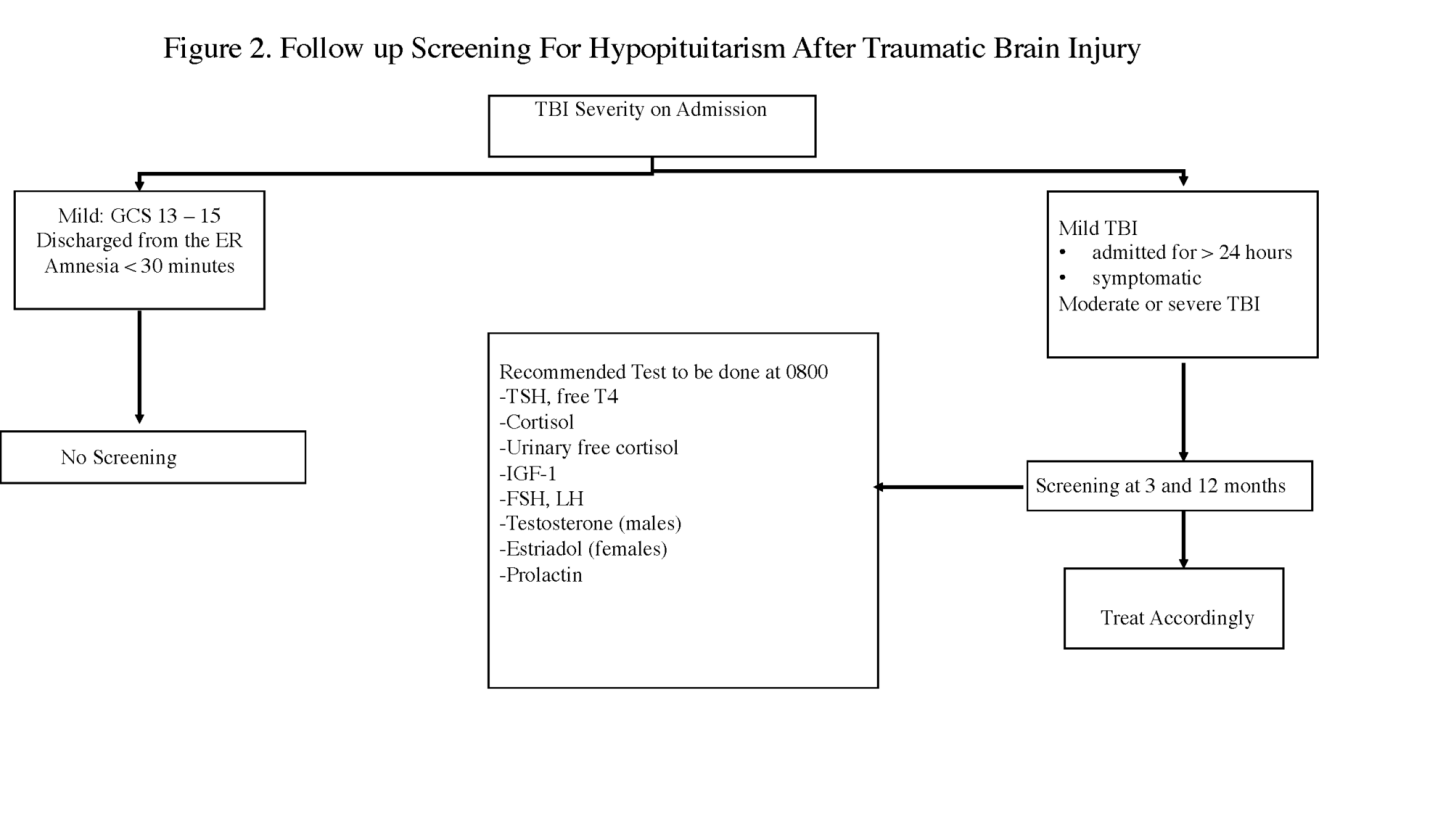
Follow-up Screening for Hypopituitarism After Traumatic Brain Injury AI - adrenal insufficiency; DAI - diffuse axonal injury; FSH – follicle stimulating hormone; GCS - Glasgow Coma Scale; ICP - intracranial pressure; IGF-1 -  insulin-like growth factor 1; LH - luteinizing hormone; TBI - traumatic brain injury; TSH - thyroid stimulating hormone; ug/dl - microgram per deciliter

To summarize, TBI patients who required hospitalization for at least 24 hours, those with an abnormality on the initial head computed tomography (CT), and those who have signs and symptoms of pituitary failure after TBI should be screened at three months and one year post-injury and even further out if symptomatic (Figure 2). All symptomatic patients should be screened immediately. Consultation with an endocrinologist is recommended for added expertise and the management of hormone replacement therapy if required. 

Predictors of neuroendocrine dysfunction

In a retrospective study, Silva et al. [27] showed that post-traumatic seizures, intracranial hemorrhage, petechial brain hemorrhage, and focal cortical contusion are predictors of central adrenal insufficiency. Motor vehicle crash (MVC), as a mechanism for TBI, was a risk factor for both thyroid stimulating hormone (TSH) and ACTH deficiencies. Hospitalization after TBI and the presence of intracranial hemorrhage on imaging showed trends towards being risk factors for GH deficiency. Other conditions associated with hypopituitarism are advanced age, increased intracranial pressure, prolonged intubation greater than 10 days, diffuse axonal injury, basal skull fractures, hypoxia, hypotension, and the duration of coma. Cranial vault fracture was found to correlate negatively [22, 27-28]. In a study by Schneider et al., patients with DI were primarily victims of MVCs and had brain MRI showing intracranial hemorrhage, subarachnoid hemorrhage, or focal cortical contusion [28]. They concluded that TBI patients who have experienced an MVC and/or those with post-traumatic seizures, intracranial hemorrhage, petechial brain hemorrhages, and/or focal cortical contusions are at high risk for neuroendocrine dysfunction, including central adrenal insufficiency and DI, and should be referred for pituitary hormone testing. The predictors appear to be applicable to most TBI patients and hence, the broad recommendation to screen most patients.

The Endocrine Society Clinical Practice Guidelines published in 2016 provides recommendations for the diagnosis of hypopituitarism [29]. The guideline recommends measuring serum cortisol levels at 8 - 9 am as the first-line test for diagnosing central adrenal insufficiency (AI). A cortisol level < 3 μg/dL is indicative of AI and a cortisol level > 15 μg/dL likely excludes an AI diagnosis. A corticotropin stimulation test can be performed to diagnose AI when the morning cortisol values are between 3 μg/dLand 15 μg/dL. Peak cortisol levels < 18.1 μg/dL at 30 minutes or 60 minutes indicate AI. For central hypothyroidism, a free T4 level below the laboratory reference range in conjunction with a low, normal, or mildly elevated TSH in the setting of pituitary disease is usually confirmatory. For GH deficiency (GHD), GH stimulation testing is recommended as single GH measurements are not helpful. Appropriately controlled body mass index (BMI) cutoffs to assess peak GH values should be done. Biochemical testing for GHD in patients with clear-cut features of GHD and other documented pituitary hormone deficits should not be done. The stimulation test can be the insulin tolerance test, the glucagon stimulation test, the GH-releasing hormone (GHRH) arginine test, or GHRH/GH releasing peptide-6 (GHRP-6) test. The GHRH/GHRP-6 test is noted to be reliable and is not affected by factors which modify GH secretions. In males with suspected hypogonadism, it is recommended to measure the serum testosterone, FSH, and LH in the absence of acute/subacute illness and before 10 am (after an overnight fast) combined with a serum prolactin (PRL) level. In the presence of oligomenorrhea or amenorrhea, it is recommended to measure serum estradiol (E2), FSH, and LH. Clinicians should exclude other causes of menstrual irregularities related to impaired ovulation. For postmenopausal women, the absence of high serum FSH and LH is sufficient for a diagnosis of gonadotrope dysfunction (provided the patient is not on hormone replacement therapy (HRT)). To assess for central diabetes insipidus, serum and urine osmolarity should be measured simultaneously in patients with polyuria (more than 50 mL/kg of body weight/24 hours, 3.5 liters/day (L/d) in a 70-kg person). In the presence of high serum osmolarity (> 295 mOsmol/L), urine osmolarity should reach approximately 600 mOsmol/L (urine osmolality/plasma osmolality ratio should be ≥ 2), whereas the urine dipstick should be negative for glucose. The involvement of an endocrinologist is recommended to ensure that appropriate testing and follow-up is provided [29]. In addition, hospital discharge information should include the recommendation for the screening by the patient’s primary care provider.

In the chronic phase, post-TBI hypopituitarism can manifest as lethargy, poor sleep, inattention, difficulty concentrating, memory impairment, anxiety, poor judgment, depression, irritability, insomnia, and diminished libido [23]. Wachter et al. [30] found that neuropsychological and quality of life (QoL) deficits were associated more frequently with hemorrhagic lesions on CT scans than with hypopituitarism. 

Management and outcomes

Hypopituitarism contributes to TBI-related mortality and functional and cognitive morbidity. GH deficiency has the most effect on the outcome [31-32]. There is a clear association between pituitary dysfunction and adverse cognitive outcomes, such as memory, attention, language, physical conditioning, and mood disorders. It can also lead to dyslipidemia, hypertriglyceridemia, adiposopathy, insulin resistance, reduced quality of life, and increased risk of premature cardiovascular death from endothelial dysfunction and atherosclerosis [33]. These findings are independent of the BMI of the patient. It may also limit recovery and rehabilitation [34], leading to chronic disability. There is reported improvement in the QoL after GH replacement in post-TBI hypopituitarism, as well as improvement in cognitive impairments and functional independence measures. Adrenal crisis after TBI can be treated with glucocorticoid replacement [35-41]. Testosterone replacement in hypogonadal men is associated with decreased anger and irritability and increased libido and energy. Replacement of estrogen has been shown to improve verbal memory, reasoning, vigilance, and motor speed in symptomatic postmenopausal females. Patients with post-TBI pituitary dysfunction may receive suboptimal rehabilitation unless the underlying hormone deficiency is identified and treated. Replacement is also important in these post-traumatic patients who maybe require further surgical intervention and are at risk for adrenal insufficiency during the postoperative period. Once identified, TSH, antidiuretic hormone (ADH), and ACTH deficiencies should be replaced, but GH replacement therapy in adults in the setting for hypopituitarism is controversial. Therefore, referral to an endocrinologist is recommended for individualized management of the various deficiencies. 

## Conclusions

Clinicians involved in the management of TBI patients should consider hypopituitarism and its impact on long-term morbidity. Evaluation of pituitary function on an ongoing basis after TBI is paramount irrespective of the severity of the initial injury. Endocrinology and physiatry involvement for specialized testing and long-term follow-up is recommended for all TBI patients who were hospitalized and/or are symptomatic. Hormonal replacement is essential for optimal rehabilitation for TBI patients with a positive screen. Continuing medical education will increase physician awareness, and informing patients and their families will help to foster better follow-up. Further studies are needed to determine the true incidence of hypopituitarism, define the pathophysiology of hypopituitarism after TBI, and refine the predictors of hypopituitarism and the effects of pituitary hormone replacement on the metabolic profile, psychosocial issues, and neurocognitive behaviors.
